# Bevacizumab and intraocular tumors: an intriguing paradox

**Published:** 2012-10-05

**Authors:** Mariam el Filali, Long V. Ly, Gregorius P.M. Luyten, Mieke Versluis, Hans E. Grossniklaus, Pieter A. van der Velden, Martine J. Jager

**Affiliations:** 1 Department of Ophthalmology, LUMC, Leiden, the Netherlands; 2 Department of Ophthalmology, Emory University School of Medicine, Atlanta, GA; 3 Department of Pathology, Emory University School of Medicine, Atlanta, GA

## Abstract

**Purpose:**

Bevacizumab, a humanized monoclonal antibody to vascular endothelial growth factor-A (VEGF-A), was originally developed as an anti-tumor treatment. In ocular oncology, it is being used to treat macular edema due to radiation retinopathy, but it may also be useful for the treatment of primary uveal melanoma (UM) or its metastases. We determined the effect of bevacizumab on the growth of B16F10 cells inside the eye and on B16F10 and UM cells cultured in vitro.

**Methods:**

B16F10 melanoma cells were placed into the anterior chamber of the eye of C57Bl/6 mice and tumor growth was monitored after injection of different doses of bevacizumab or mock injection. In addition, the effect of bevacizumab on in vitro growth of B16F10 and human UM cells and on the expression of VEGF-A, GLUT-1, and HIF-1α was evaluated.

**Results:**

Following intraocular injection of bevacizumab into murine B16 tumor-containing eyes, an acceleration of tumor growth was observed, with the occurrence of anterior chamber hemorrhages. Bevacizumab did not affect proliferation of B16F10 cells in vitro, while it inhibited UM cell proliferation. Expression analysis demonstrated that addition of bevacizumab under hypoxic conditions induced VEGF-A, GLUT-1 and HIF-1α in B16F10 cells as well as in UM cell lines and two of four primary UM tumor cultures.

**Conclusions:**

In contrast with expectations, intraocular injection of bevacizumab stimulated B16F10 melanoma growth in murine eyes. In vitro exposure of B16 and human UM cells to bevacizumab led to paradoxical VEGF-A upregulation. The use of VEGF inhibitors for treatment of macular edema (due to radiation retinopathy) after irradiation of UM should be considered carefully, because of the possible adverse effects on residual UM cells.

## Introduction

Uveal melanoma (UM) is the most common primary intraocular tumor in adults with an annual incidence of 7–10 cases per million per year. Current treatments of UM depend on several clinical factors and include enucleation, radiotherapy (plaque, proton beam or stereotactic irradiation), transpupillary thermotherapy (TTT) and local resection [1-7]. Radiotherapy using a radioactive plaque is a highly successful therapy, achieving local tumor control of UM in up to 97% of treated cases [8-10]. However, radiation therapy may lead to radiation retinopathy, a slowly progressive, delayed-onset disease of retinal blood vessels characterized by retinal ischemia, neovascularization and leaking vessels [11-13]. Radiation retinopathy has been described in up to 63% of eyes after plaque radiation treatment [14-17]. Vascular endothelial growth factor (VEGF)-A, a strong pro-angiogenic factor, most likely contributes to its pathogenesis: it has been shown that VEGF-A can be produced by retinal tissue as well as hypoxic UM cells [18,19]. Intravitreal bevacizumab (a humanized monoclonal antibody to VEGF-A) has been used to treat numerous ophthalmologic disorders involving ischemia and neovascularization, including radiation retinopathy. Several studies demonstrate a decrease of macular edema in radiation retinopathy and improvement of visual acuity after intravitreal bevacizumab treatment [20-26].

Another indication for using bevacizumab might be the treatment of the tumor itself and its metastases. Bevacizumab has been approved for treatment of metastases of several malignancies, including colorectal, renal, and lung cancers [27-29], and is still under investigation for numerous other primary tumors and metastatic disease, e.g., of breast and pancreas cancer and cutaneous melanoma [30-32]. Despite the good primary tumor control achieved by current treatment options, estimates of 5-year UM-related mortality range from 26% to 32% [33,34], and up to 50% of all UM patients may eventually die due to metastatic disease [35,36].

Blood vessels in primary UM can facilitate tumor cell entry into the circulation, resulting in metastatic disease [37]. Yang et al. studied the systemic treatment of metastatic disease of UM with bevacizumab in mice and demonstrated a reduction in the number of metastases [38]. There are no studies describing a possible treatment with intravitreal VEGF inhibitors for primary uveal melanoma. It has been proposed that patients who develop clinical metastases from UM often harbour micrometastases for years which will most likely resemble the primary UM cell genotype [39]. Also, following radiation therapy of an intraocular melanoma, viable UM cells may remain, and these might be influenced by intraocular treatment with intravitreal bevacizumab. While bevacizumab might be a useful drug to attack uveal melanoma, several studies have been published describing unexpected effects of bevacizumab on tumor cells, resulting in tumor recurrences and therapy resistance [40,41]. We therefore investigated the effect of bevacizumab on intraocular tumor growth of the murine B16F10 melanoma cell line in an in vivo mouse model, and analyzed its effect on proliferation of this B16F10 cell line, on UM cell lines in vitro and on primary UM cell cultures [42].

## Methods

### In vivo experiments

Male C57BL/6jico mice, 8 weeks old, were obtained from Charles River (France). The mice were housed under Specific Pathogen Free (SPF) conditions and cared for in accordance with the guidelines of the University Committee for the Humane Care of Laboratory animals, NIH guidelines on laboratory animal welfare, and the ARVO statement for the Use of Animals in Ophthalmic and Vision Research. All research protocols were approved by the local Committee for Animal Welfare (DEC), LUMC, Leiden, The Netherlands. B16F10 melanoma cells were injected into the anterior chamber of the mice as described previously [43]. In short, mice were anesthetized intraperitoneally using Xylazine (Rompun 2%, Bayer, Leverkussen, Germany) and Ketamine Hydrochloride (Aescoket, Aesculaap bv, Boxtel, the Netherlands; ratio 1:1). A sterile 30 G needle was used to make a paracentesis at the corneoscleral junction, parallel and anterior to the iris. A fused silica capillary (200 μm outer diameter (OD), 100 μm inner diameter (ID) was fitted into a union (VALCO, Vici AG international, Schenkon, Switzerland). The capillary and the union were mounted onto a 0.1 ml Hamilton syringe. The capillary, loaded with B16F10 melanoma cells (2.5. 10^4^ cells/ 4 μl (approximate volume of anterior chamber of murine eye), was inserted through the paracentesis in the cornea, and the tumor cells were deposited into the anterior chamber.

Mice were separated into three groups (n=7 per group) and the experiment was performed twice; the first group received a low dose of bevacizumab (equivalent human dose; 2µg/4 µl), the second group received a high dose (10 times the equivalent human dose; 20 µg/4 µl) of bevacizumab; a control group was injected with a mock injection with 4 µl phosphate-buffered saline (PBS). The eyes were examined three times a week using a dissecting microscope to monitor tumor growth. Tumor volume was recorded as the percentage of anterior chamber occupied with tumor. Mice were sacrificed by cervical dislocation when the tumor occupied 80%–100% of the anterior chamber. Afterwards, all murine eyes were

fixed in 10% neutral-buffered formalin for 48 h and embedded in paraffin. Serial sections of 4 μm were prepared, mounted on glass slides and stained with hematoxylin and eosin. The amount of vessels and their location were evaluated in tumor-containing eyes of all three groups of one of the two experiments (n=7 per group).

### Cell lines and primary tumor cell cultures

The B16F10 melanoma cell line was cultured in Iscove's Modified Dulbecco's Medium (IMDM; Invitrogen, CA), supplemented with 10% fetal calf serum (FCS), glutamine and 2% penicillin/ streptomycin. Cells were incubated at (37 °C, 5% CO_2_). When cultures showed 70% confluency, the cells were harvested and used for inoculation or in vitro experiments.

Two UM cell lines and four primary UM cell cultures were cultured under either normoxic (20% O_2_) or hypoxic (1% O_2;_ during experiments) conditions (HERAcell 240 CO_2_ Incubator, Thermo Fisher Scientific Inc.). Mel 285 is a primary tumor-derived cell line, while OMM2.3 is a metastasis-derived cell line. Mel285, and OMM2.3 were provided by dr. B. Ksander (Schepens Eye Research Institute, Boston, MA) [44,45]. Four primary cell cultures were established in our laboratory. UM cell lines were cultured in Dulbecco’s Modified Eagle’s Medium (DMEM) supplemented with 10% fetal calf serum and 1% penicillin/streptomycin (GIBCO, Life Technologies, Paisley, UK). The cells were passaged once or twice a week using trypsin (0.05%). Fresh tumor tissues, obtained immediately after enucleation of the eye, were cultured in Amniochrome^®^ Pro Medium (Lonza Group Ltd, Basel, Switzerland) and passaged maximally once or twice before experiments.

### Cell proliferation

 Cell proliferation was measured by mitochondrial function using the water-soluble tetrazolium salt (WST-1) assay (Roche Diagnostics, Indianapolis, IN), as previously described [46]. In short, 96-well plates were filled with 1200 cells per well, filled with regular medium (control) or bevacizumab solutions (three doses, see ‘bevacizumab treatment’ on the next page), and either placed in a normoxic (20% O_2_, 5% CO_2_, 37 °C) or hypoxic (1% O_2_, 5% CO_2_, 37 °C) chamber to mimic in vivo ischemia (HERAcell 240 CO_2_ Incubator, Thermo Fisher Scientific Inc.). Proliferation was measured on days 1, 2, and 3 (B16F10) or 1, 3, and 6 (human cell lines and primary cultures) after adding bevacizumab by adding 10ul WST-reagent to 8 wells (8 wells without reagent for background absorbance). Absorbance was measured at 450 nm (n=8) on a multiwell spectrophotometer (Perkin lmer, Wellesley, MA).

### Quantitative PCR

To analyze VEGF-A mRNA expression after treatment with bevacizumab, reverse transcriptase in combination with quantitative polymerase chain reaction (qPCR) experiments were performed, as described before [19,47]. In short, RNA was isolated using an Rneasy® Mini Kit (Qiagen, Valencia, CA) and RNA samples were reverse-transcribed using the iScript cDNA synthesis kit (Bio-Rad, Hercules, CA). Using 96-well plates, a solution of sample cDNA, iQ SYBR Green Supermix, forward and reverse primers for beta-actine (β-ACTIN), ribosomal protein S11 (RPS-11), VEGF-A and sterile water was prepared. Primer sequences were used as described previously [19,48] A quantitative analysis of the samples was then performed for gene expression by qPCR in a IQ5 PCR system (Bio-Rad). The PCR reaction settings were 95 °C for 3 min, 40 cycles at 95 °C for 30 s and 60 °C for 30 s, followed by 95 °C for 1 min, and 60 °C for 1 min. To correct the sample-to-sample variation, several cellular housekeeping genes were selected using Genorm software (Center for Medical Genetics, Ghent, Belgium), and these served as an endogenous control against which the target-gene expression levels were normalized (normalized expression of treated cells/ normalized expression of control cells after 24 h) [47-49].

### VEGF-A protein expression

The VEGF-A protein concentration was measured in the supernatant of the B16F10 cell culture using a commercial sandwich enzyme-linked immunosorbent assay (mouse VEGF-A ELISA, Arcus Biologicals, Modena, Italy). The lowest measurable concentration was 4.5 pg/ml.

### **HIF-1α** **in-cell Western experiment**

To determine bevacizumab influences VEGF-A expression in UM cells through the HIF-1α pathway, phosphorylation and activation of HIF-1α were analyzed using an in-cell Western immunofluorescent assay as described before [19,50,51]. Briefly, 96-well plates were filled with 600 melanoma cells per well, filled with regular medium (control) or bevacizumab solutions (three doses, see ‘bevacizumab treatment’ on the next page), and placed in a hypoxic (1% O_2_, 5% CO_2_, 37 °C) chamber (HERAcell 240 CO_2_ Incubator, Thermo Fisher Scientific Inc.) for 24 h, to mimic hypoxic in vivo conditions and stimulate VEGF-A expression. Cells were fixed and permeabilized for 1.5 h before incubation with HIF-1α antibody (Bethyl laboratories INC Montgomery, TX;1:1000 for 2 h) in Odyssey Blocking Buffer. After washing, plates were incubated with the secondary antibody Goat anti-Rabbit IRDye® 800CW (1:800); for determining the cell number, DRAQ5 (1:2000) and Sapphire700 (1:1000) were used (all three from LI-COR Biosciences, Lincoln NB). The plate was scanned on an Odyssey infrared scanner, HIF-1α at the 800 nm channel and DRAQ5 at the 700 nm channel (LI-COR Biosciences). Data were acquired using the scanner software, exported to Excel (Microsoft, Redmond, WA), and analyzed by GraphPad Prism (GraphPad Software, San Diego, CA).

### Bevacizumab treatment

Several bevacizumab (Avastin®, Roche Pharma, Germany) solutions were used to investigate treatment options in vitro and in vivo.

#### In vitro

To treat UM cell cultures, bevacizumab was dissolved in medium to obtain solutions of 1.25 mg/5.46 ml (generally used intraocular human dose), 12.5 mg/5.46 ml (10 times the normal dose) or 25 mg/5.46 ml (20 times the normal dose) per well. As control, we used culture medium.

#### In vivo

Equivalent mouse doses were calculated according to human and mouse intraocular volume: as 1.25 mg bevacizumab is used to inject the human eye with a mean volume of 5.46 ml, we used 2 µg (first group; equivalent human dose) or 20 µg (second group 10 times equivalent human dose) to inject a murine eye with a mean volume of 9.60 µl. The third group received a mock injection with PBS as previously described. Injections were performed on days 2, 6, and 10 after tumor cell inoculation (not all mice received an injection on day ten since some of them were already sacrificed).

### Statistical analysis

Statistical analysis was performed with Graphpad Prism software (GraphPad Software, Inc., La Jolla, CA). Overall survival of mice was estimated by the Kaplan–Meier method and log-rank test. One-way ANOVA and the Bonferonni test were used to analyze for significant differencences in cell proliferation and HIF-1α activation of treated and untreated cells. p<0.05 was considered statistically significant.

## Results

### Intraocular tumor growth in a B16F10 eye tumor mouse model

**Figure 1:**
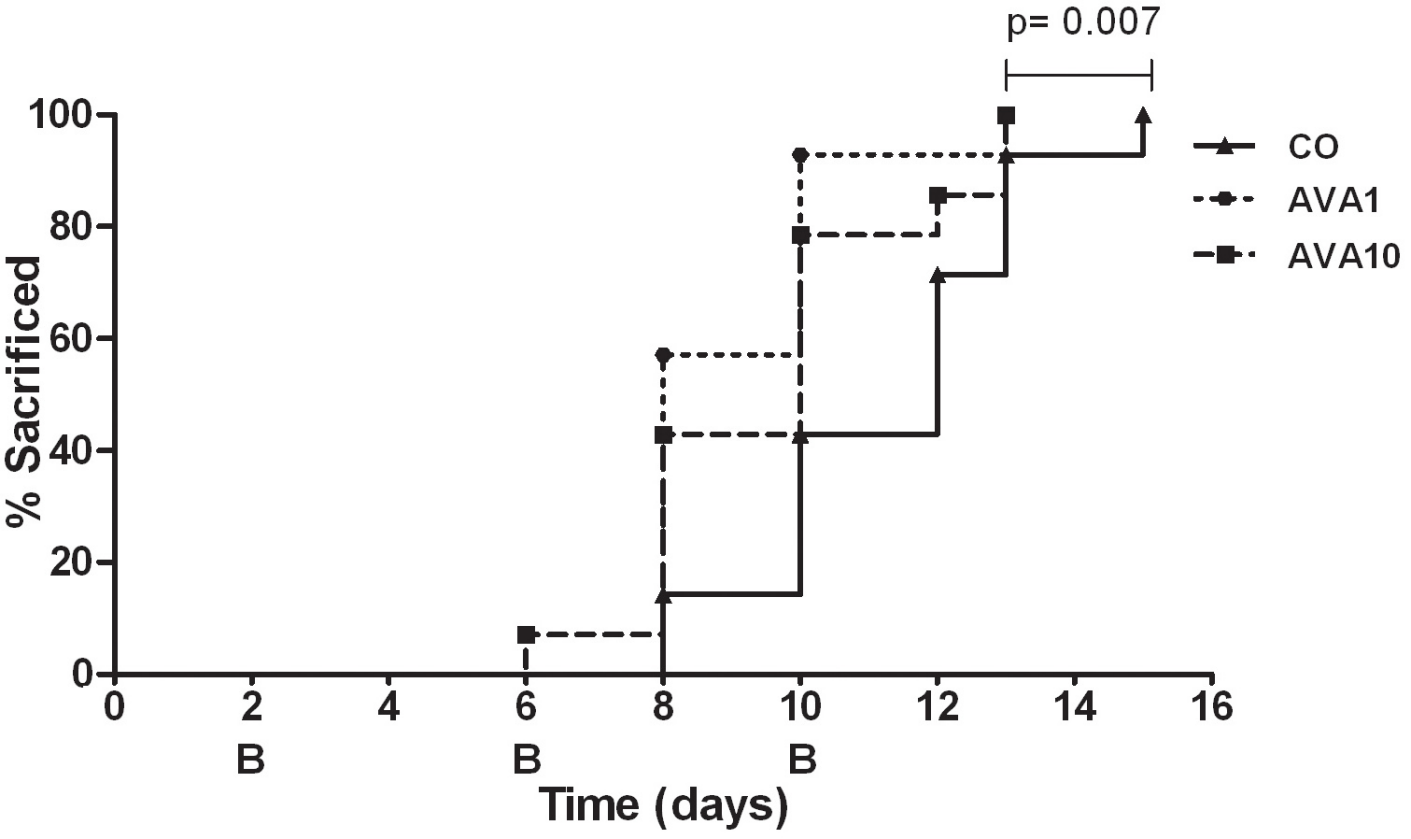
Tumor growth after bevacizumab treatment**.** After placement of B16F10 melanoma cells in the eye of C57Bl/6 mice, bevacizumab was injected intraocularly to try to inhibit intraocular tumor growth. Following three bevacizumab injections on days 2, 6, and 10, a significant acceleration of intraocular tumor growth occurred compared to the control group (AVA1 versus control p=0.007, and AVA10 versus control p=0.06). Growth was recorded as the percentage of anterior chamber occupied with tumor, and mice were sacrificed when the tumor occupied 80%–100% of the anterior chamber. The curves are the pooled data from two experiments, with 14 mice in each of the three groups. AVA1=equivalent human dose: 2 μg/4 µl; AVA10=10 times the equivalent human dose: 20 μg/4 μl; CO=control group: 4 µl mock PBS injection.

**Figure 2:**
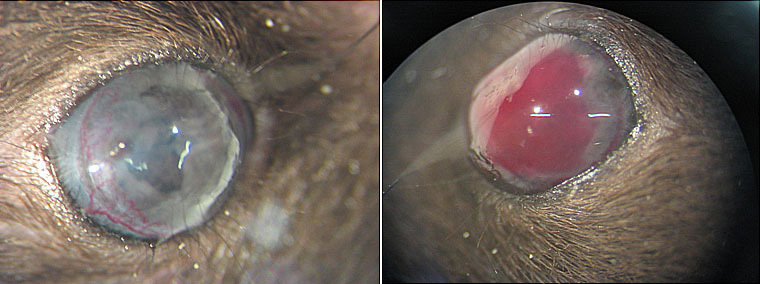
Anterior chamber of murine eyes treated without or with bevacizumab. Anterior chamber hemorrhage as seen in a murine tumor eye treated with bevacizumab (right) in contrast to an untreated eye (left).

As VEGF-A inhibitors are being used to treat many different types of cancer metastases, we wondered whether they can be used to inhibit intraocular tumor growth. After intraocular inoculation of B16F10 melanoma cells into the anterior chamber of C57Bl/6 mice, two different doses of bevacizumab or medium were injected intraocularly on days 2, 6, and 10. Each group consisted of seven mice, and the experiment was performed twice. A summary of the results of the two experiments is shown in Figure 1. Whereas inhibition of tumor and vessel growth was expected, an acceleration of intraocular tumor growth was observed in eyes treated with both doses of bevacizumab. Treated mice had to be sacrificed earlier because the tumor started to protrude through the cornea. During the experiment, several anterior chamber and tumor hemorrhages were observed in the eyes that had been treated with bevacizumab but not in untreated eyes (Figure 2).

### B16F10 murine melanoma cell line: in vitro experiments

#### In vitro cell proliferation

**Figure 3:**
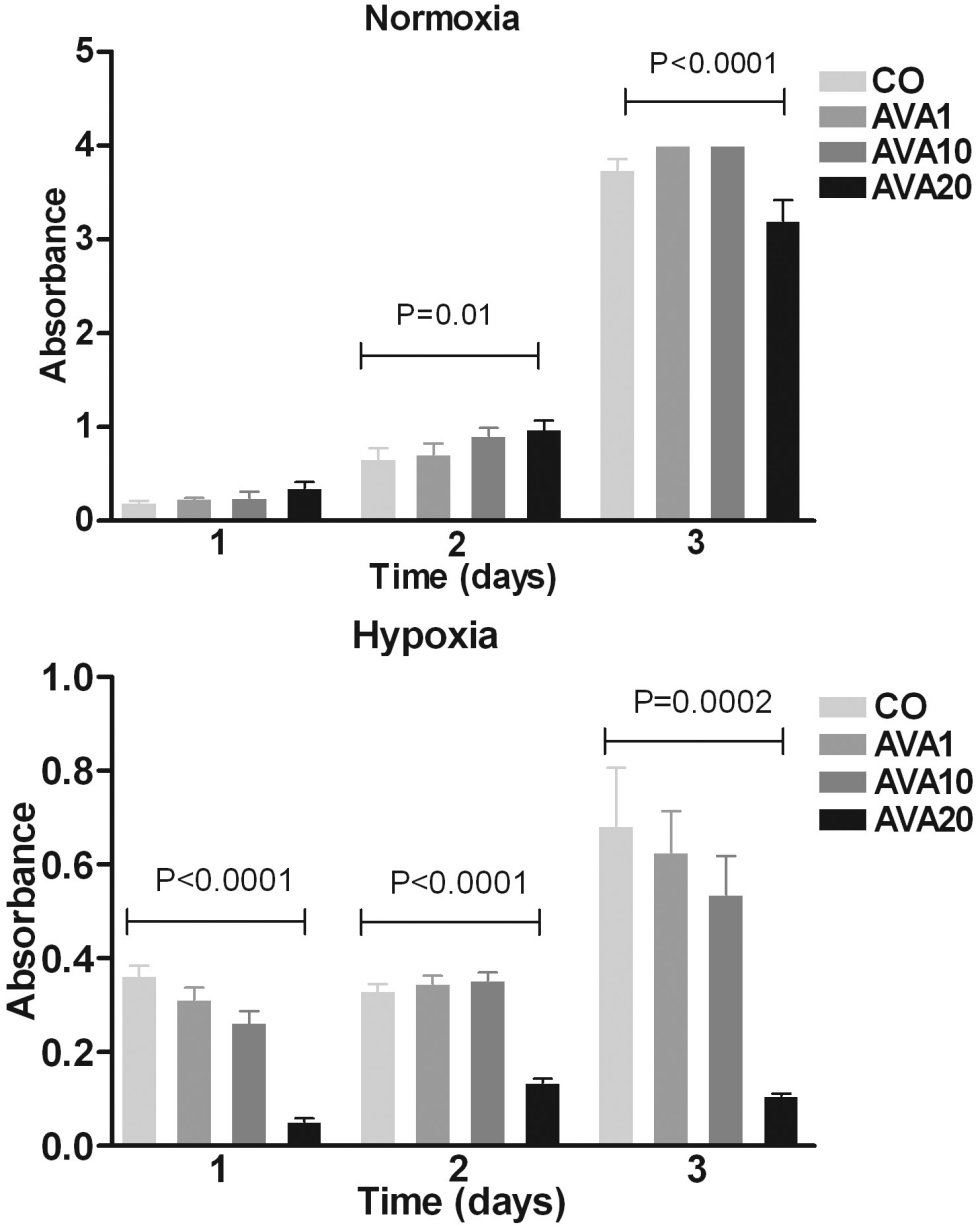
Proliferation of B16F10 cells after bevacizumab treatment. Bevacizumab was added to B16F10 cells in vitro under normoxic (top) and hypoxic (bottom) circumstances and proliferation was measured by WST-1 assay on days one, two and three after addition of bevacizumab. Cell density is expressed in absorbance (optical density, OD). A high dose of bevacizumab inhibited cell growth under hypoxic conditions.

As the in vivo results showed the opposite effects of what was expected, we studied the effect of bevacizumab on tumor cell proliferation under normoxia and hypoxia. Addition of bevacizumab to B16F10 cells in vitro did not effect proliferation of tumor cells cultured in normoxic conditions, while a dose-dependent decrease in proliferation was noticed after treatment with bevacizumab under normoxic conditions (Figure 3).

#### In vitro VEGF-A mRNA expression

**Figure 4:**
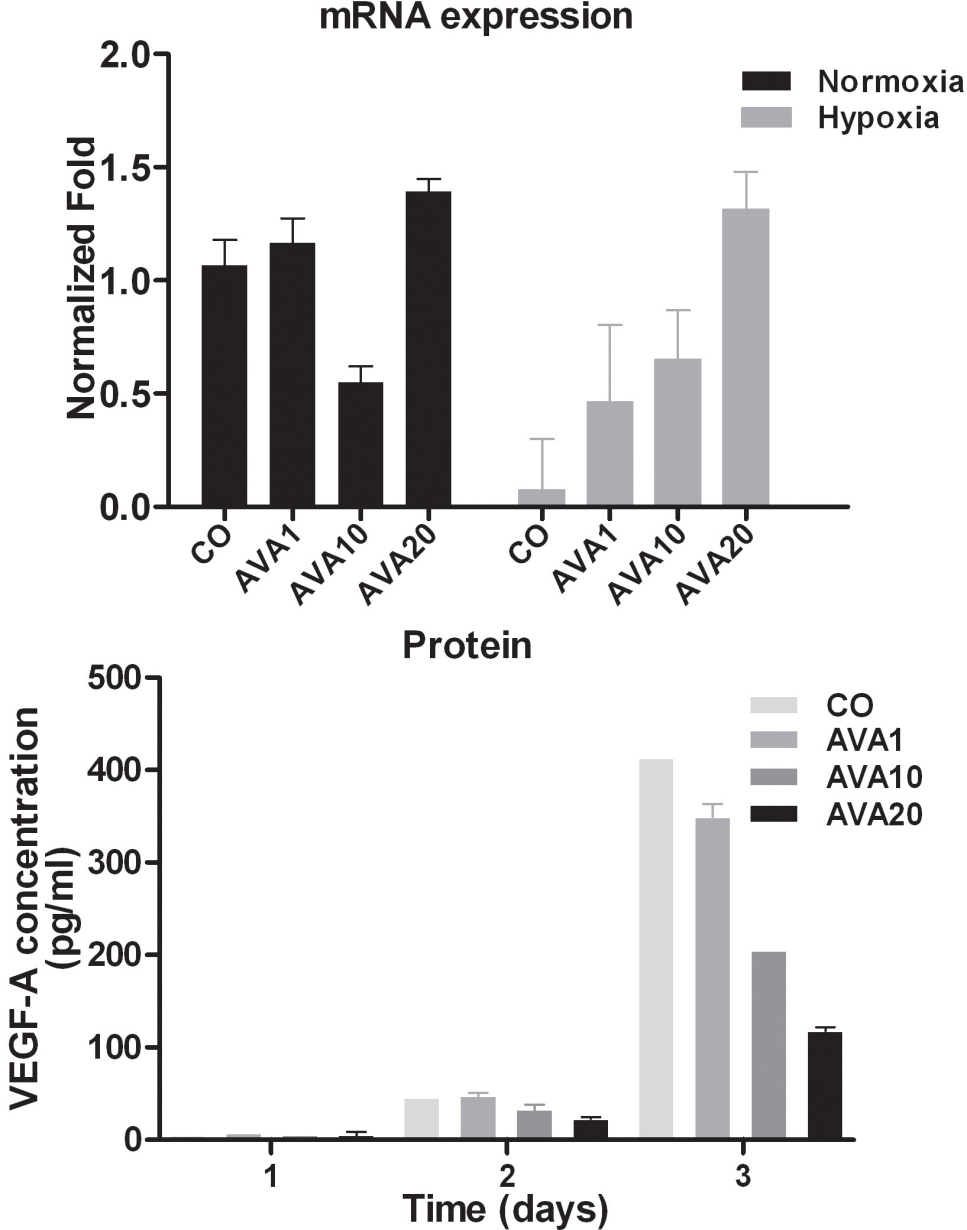
VEGF-A expression of B16F10 cells after bevacizumab treatment The effect of bevacizumab on VEGF-A expression in B16F10 cells was determined using qPCR analysis (top) and ELISA (bottom). The amount of VEGF-A mRNA expression was measured with qPCR under normoxic (black) and hypoxic (gray) conditions after 24 h with different doses bevacizumab treatment. Expression is demonstrated in normalized fold. VEGF-A mRNA expression increased in a dose-dependent manner after treatment with bevacizumab, when cells were cultured under hypoxic conditions. An ELISA was used to measure the amount of VEGF-A protein in the supernatant of UM cell lines after hypoxic exposure for 24 h. Shown is the amount of VEGF-A protein (pg/ml). Paradoxically, the amount of produced protein is reduced in the presence of bevacizumab. AVA1=equivalent human dose: 1.25 mg/5.46 ml; AVA10=10 times the equivalent human dose: 12.5 mg/ 5.46 ml; AVA20=20 times the equivalent human dose: 25 mg/5.46 ml; CO=control group: culture medium.

As intraocular and intratumoral hemorrhages were visible in eyes receiving bevacizumab, we wondered whether the anti-VEGF-A treatment with bevacizumab had a paradoxical stimulatory effect on VEGF-A gene expression. Indeed, bevacizumab induced VEGF-A mRNA expression in a dose-dependent manner when cells were cultured under hypoxic conditions. Under normoxia, baseline VEGF-A expression is relatively high but not affected by bevacizumab treatment (Figure 4).

In contrast to mRNA expression, in vitro treatment of B16F10 cells with the human equivalent dose of bevacizumab led to a lower VEGF protein expression after 3 or 6 days of culture (Figure 4). We hypothesize that the high concentration of bevacizumab effectively bound the produced VEGF-A, thus making it unavailable for binding in the immuno assay.

#### Histologic and in vivo observation of tumor-inoculated murine eyes

Based on the in vitro results, the acceleration of tumor growth in vivo could not be due to a direct proliferative effect, but possibly due to formation of new vessels after induction of VEGF-A. After sacrificing the mice, all eyes were enucleated and stained with hematoxylin and eosin to evaluate the amount of vessels and their location in the tumor. No apparent differences were present in the amount of vessels in the presence or absence of bevacizumab treatment (data not shown).

#### Involvement of HIF-1α pathway

**Figure 5:**
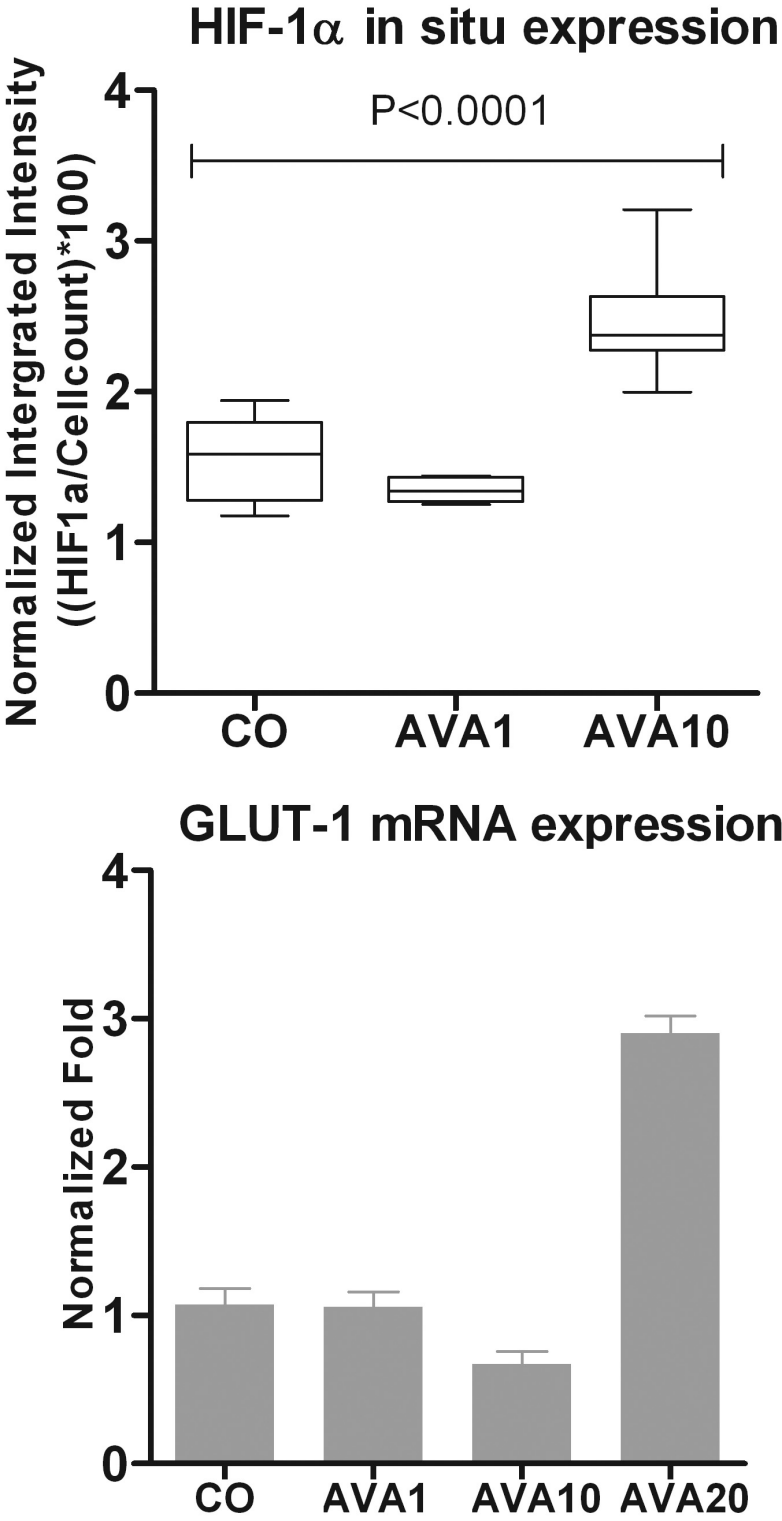
Involvement of HIF-1α in B16F10 cells after bevacizumab treatment under hypoxic conditions. Expression of HIF-1α was measured in B16F10 cells using an in-cell Western assay after cell exposure to hypoxic conditions for 24 h. Expression is demonstrated in normalized integrated intensity ((HIF-1α/cell count)*100). A significant induction of HIF-1α was observed when comparing the AVA10 treated cells with the control (p=0.03; top). qPCR analysis shows an induction of GLUT-1 expression in B16F10 cells treated with the high dose of bevacizumab (AVA20) in comparison to no treatment. Expression is demonstrated in normalized fold (bottom). AVA1=equivalent human dose: 1.25 mg/5.46 ml; AVA10=10 times the equivalent human dose: 12.5 mg/ 5.46 ml; AVA20=20 times the equivalent human dose: 25 mg/ 5.46 ml; CO=control group: culture medium.

As the new working hypothesis was that the observed intraocular tumor growth after bevacizumab was due to an increased production of VEGF-A, one might expect an upregulation of the pathways involved in VEGF-A induction. To investigate whether the increased VEGF-A mRNA expression after exposure to bevacizumab involved HIF-1α, an in-cell Western assay was performed. Under already hypoxic conditions, treatment with 10 times the human equivalent of bevacizumab induced an increase of HIF-1α protein in B16F10 melanoma cells in comparison to the control (Figure 5).

To further confirm involvement of HIF-1α, GLUT1 expression was measured as a determinant of HIF-1α transcriptional activity. Gene expression analysis showed an induction of GLUT1 expression in B16F10 cells treated with bevacizumab in comparison to control, but only when treated with the highest dose (Figure 5).

### Validation in human UM cell lines and primary cultures

#### In vitro uveal melanoma cell proliferation

**Figure 6:**
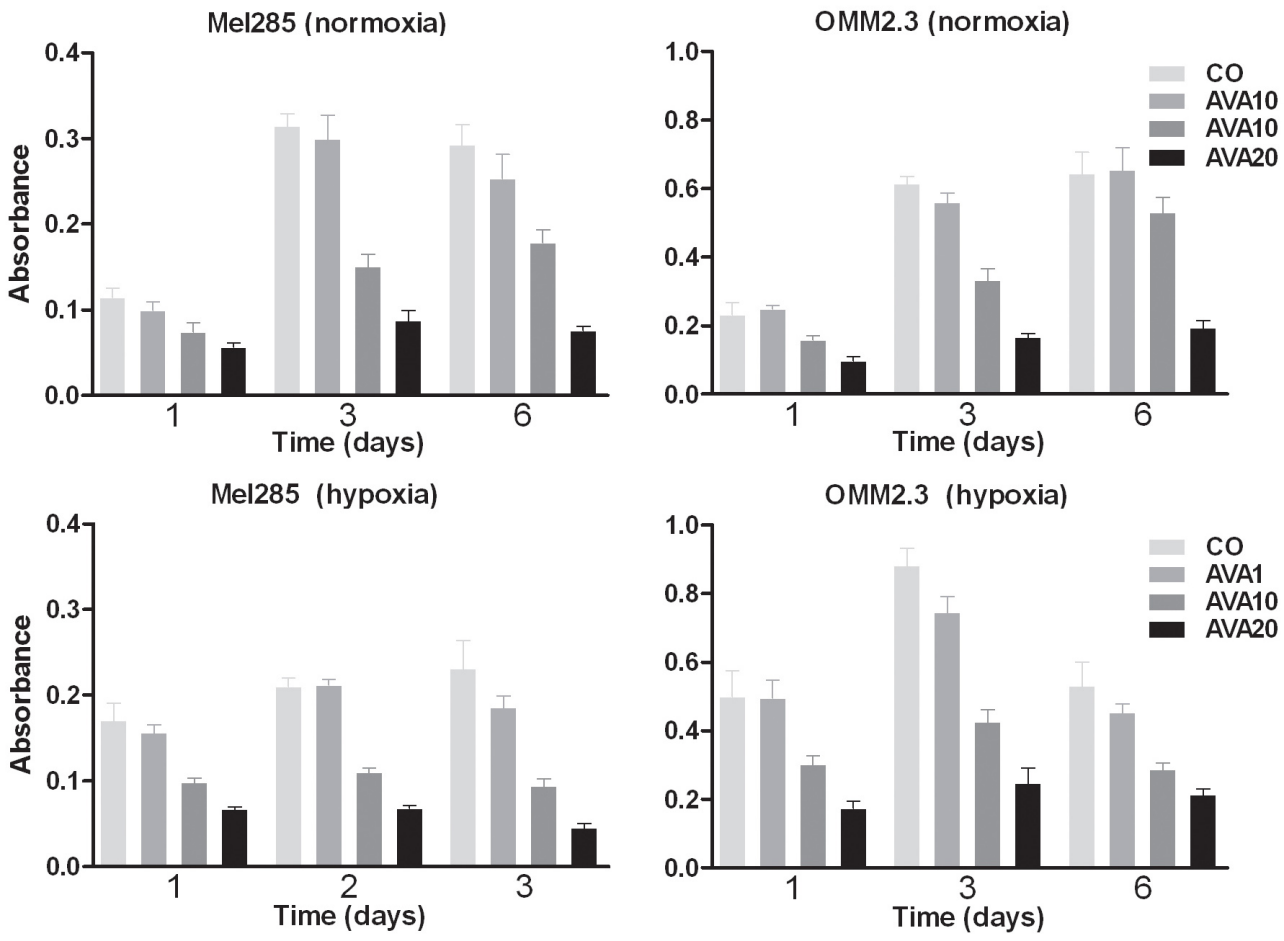
Proliferation of UM cell lines after bevacizumab treatment. Proliferation of UM cell lines was measured by WST-1 assay after exposure to three different doses of bevacizumab during one, three or six days, under normoxic and hypoxic conditions. Cell density is expressed as absorbance (optical density, OD). A dose-dependent decrease of proliferation after treatment with bevacizumab occurs under hypoxic as well as normoxic conditions. AVA1=equivalent human dose: 1.25 mg/ 5.46 ml; AVA10=10 times the equivalent human dose: 12.5 mg/ 5.46 ml; AVA20=20 times the equivalent human dose: 25 mg/ 5.46 ml; CO=control group: culture medium.

To determine whether the mechanism observed in murine B16F10 cells was relevant to the human situation, the in vitro tests were repeated using human UM cell lines and primary cell cultures. Following treatment with bevacizumab, human UM cell lines showed a dose-dependent decrease of proliferation under normoxic as well as hypoxic culture conditions (Figure 6). Four primary tumor cell cultures displayed a greater variability: in two cultures inhibition of proliferation was observed while two other cell cultures appeared resistant and did not show an effect (data not shown).

#### In vitro VEGF-A mRNA expression after bevacizumab treatment

**Figure 7:**
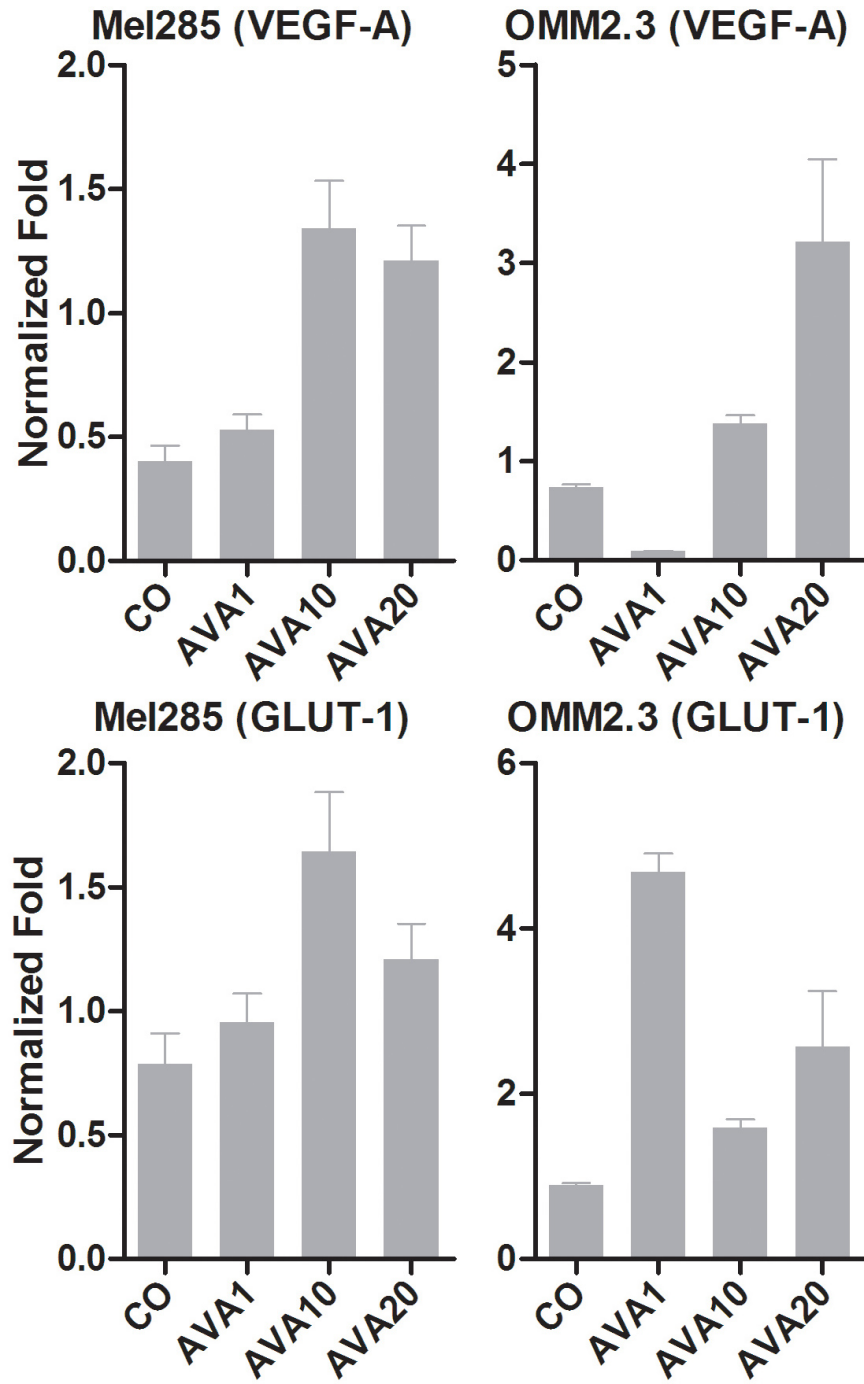
mRNA expression of UM cell lines after bevacizumab treatment mRNA expression was measured with qPCR after exposure to different doses of bevacizumab under hypoxic conditions for 24 h. Expression is shown in normalized fold. Treatment with bevacizumab induced VEGF-A and GLUT-1 mRNA. AVA1=equivalent human dose: 1.25 mg/ 5.46 ml; AVA10=10 times the equivalent human dose: 12.5 mg/5.46 ml; AVA20=20 times the equivalent human dose: 25 mg/ 5.46 ml; CO=control group: culture medium.

Treatment of two UM cell lines with bevacizumab under hypoxic conditions led to an induction of VEGF-A mRNA (Figure 7). Exposure of UM cells to bevacizumab at normal oxygen levels showed the same trend, but was less substantial (data not shown).

**Figure 8:**
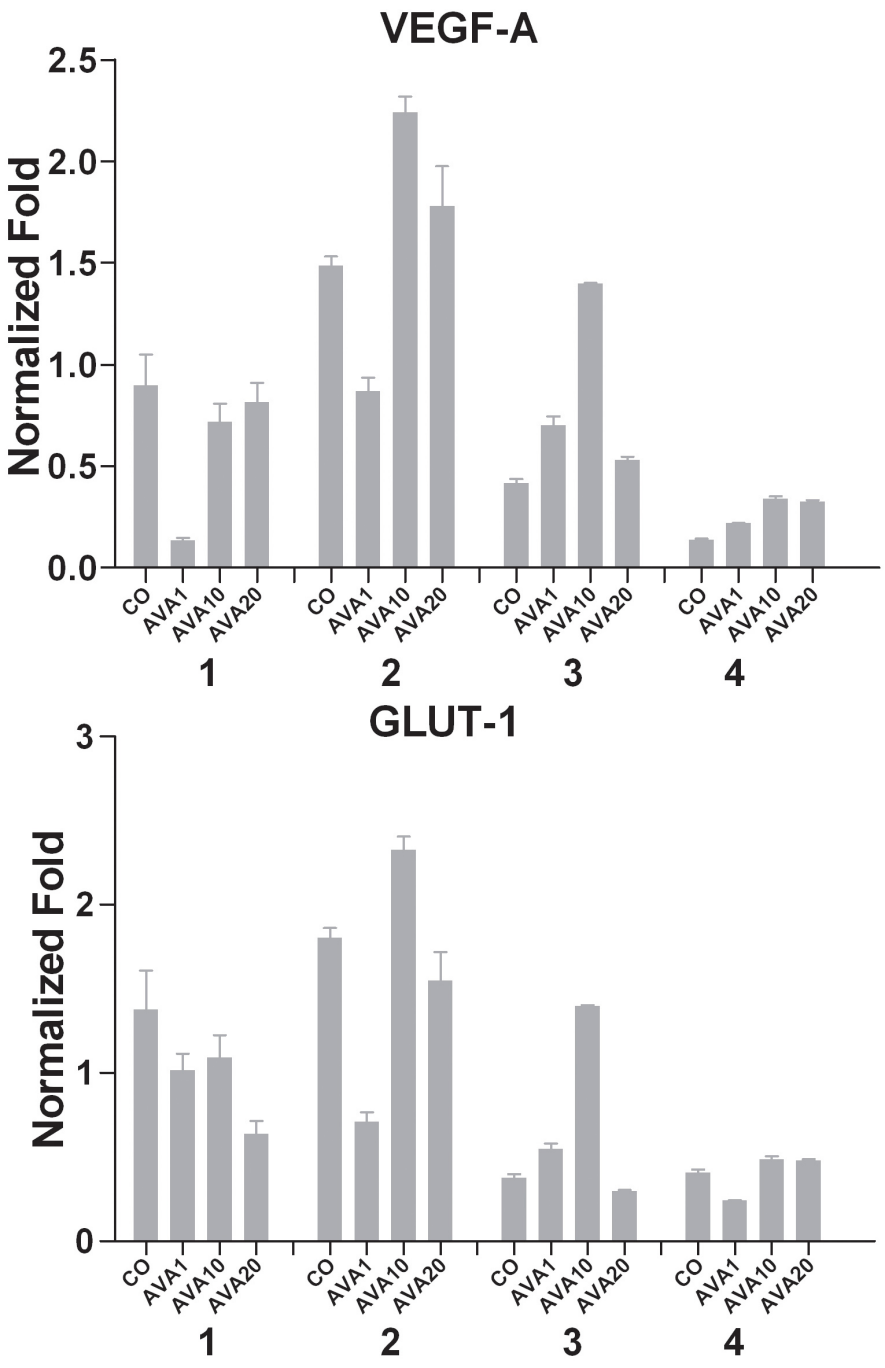
mRNA expression of UM cell cultures after bevacizumab treatment under hypoxic conditions. mRNA expression was measured with qPCR under hypoxic conditions and after 24 h of different doses bevacizumab treatment. Expression is demonstrated in normalized fold. Two cell cultures show a clear induction of VEGF-A expression after treatment with bevacizumab (culture two and three). GLUT-1 expression in UM cell cultures corresponds to the VEGF-A expression (bottom); correlation coefficient 0.974, p=0.0002. AVA1=equivalent human dose: 1.25 mg/ 5.46 ml; AVA10=10 times the equivalent human dose: 12.5 mg/ 5.46 ml; AVA20=20 times the equivalent human dose: 25 mg/ 5.46 ml; CO=control group: culture medium.

In primary UM cell cultures, the effect of bevacizumab on proliferation had been variable: in the two cell cultures which had shown reduced proliferation, bevacizumab induced VEGF-A expression (Figure 8) with a maximum fold increase of 3.36 and 2.48 (cultures two and three). The two other cultures which continued to proliferate in the presence of bevacizumab showed a high VEGF-A expression that was not affected by treatment.

#### In vitro GLUT-1 mRNA expression after bevacizumab treatment under hypoxic conditions

To confirm involvement of HIF-1α in the response to bevacizumab, GLUT1 expression was analyzed. An induction of GLUT1 expression was present following treatment of UM cell lines with bevacizumab (Figure 7). GLUT-1 expression in primary cell cultures was significantly correlated with decrease in VEGF-A expression (correlation coefficient 0.974, p=0.0002. Figure 8).

## Discussion

Anti-VEGF agents have proven to be potent therapeutic agents in ophthalmology for the treatment of Age-related Macular Degeneration (AMD), diabetic macular edema and neovascular glaucoma [47,48,52-54]. In UM, bevacizumab (Avastin®) is especially interesting because of a potential dual use: it is currently used in uveal melanoma patients to treat macular edema due to radiation retinopathy but may potentially be used to treat the primary uveal melanoma (UM) or its metastases.

We set out to determine the effect of intraocular injections of bevacizumab as a treatment for intraocular tumors in mice. Unexpectedly, an acceleration of intraocular tumor growth in response to anti-VEGF-A occurred. Previously, Yang et al. had shown a decrease of tumor size in the eye and a reduction in the number of metastases when bevacizumab was given systemically [38]. The difference in outcome may be due to a concentration effect, or it may be that direct contact with the vessel wall is important: in Yang’s study, bevacizumab reached the tumor through the intravascular route, while we treated the tumor locally.

There has been some discussion concerning whether humanized bevacizumab can effectively block and neutralize murine VEGF [55]. The sequences of human VEGF_165_ protein and mouse VEGF_164_ protein are 86% similar [56]. In addition, western blot analysis and ELISA analysis by Bock et al. demonstrated that a high dose of bevacizumab can bind human as well as murine VEGF [57,58]. Additionally, we confirmed the effects noticed in the murine cells with human UM cell lines and short-term fresh UM cultures: bevacizumab induced an upregulation of VEGF-A mRNA expression in B16F10 as well as human UM cells. This was especially true when the cells were cultured under hypoxic conditions, suggesting that a certain level of ischemia is needed to induce the VEGF-stimulating effect of bevacizumab.

We observed a relatively high VEGF-A expression under normoxic conditions in the B16F10 melanoma cells which suggests some kind of HIF-1α stabilization. This could involve the von Hippel-Lindau tumor suppressor (pVHL) that regulates the stability of Hypoxia-Inducible Factor (HIF)-1α. Loss of pVHL function results in constitutive activation of HIF-1α and thus VEGF-A expression [59,60]. Nonetheless, the observed induction of HIF-1α as well as GLUT-1 expression in response to hypoxia suggests that HIF-1α regulation is still intact.

One possible explanation for the in vivo tumor acceleration of tumor growth could be vessel formation after VEGF-A induction. Surprisingly, we detected no difference in the number of vessels between the different treatment groups. We did, however, perceive several anterior chamber and tumor hemorrhages in murine eyes treated with bevacizumab that may be a result of the VEGF-A induction. Originally, VEGF-A was referred to as vascular permeability factor (VPF) [61]. A rapid increase in vascular permeability occurs when the microvasculature is exposed acutely to any number of vascular permeabilizing factors, like VEGF-A, allowing for the diffusion of trophic substances to adjacent tumor cells [62]. An alternative explanation may be that mice were monitored for macroscopic tumor growth for merely eleven days, which may be too short for vessels to form. Moreover, the intraocular tumor is limited by the size of the anterior chamber of 2 mm which is about the limit to which cells can be supplied with oxygen and energy through the diffusion of trophic substances.

In our in vitro experiments, anti-VEGF-A therapy increased the amount of VEGF-A, mimicking hypoxic circumstances, and possibly inducing ‘pseudohypoxia’. This phenomenon has been described before in other tumors. Verhoeff et al. for example describe that VEGF inhibition by local deposition of pegaptanib decreased tumor hypoxia (GLUT-1 positive tumor cells) in murine intracerebral glioma [63]. This ‘pseudohypoxia’ subsequently can activate alternative pro-angiogenic signaling circuits, as was noticed in a murine pancreatic neuroendocrine cancer model. In this model, treatment with an antibody that specifically blocked VEGFR signaling caused an initial response of tumor stasis followed by tumor recurrence afterwards. The relapsing tumor expressed higher levels of mRNAs of pro-angiogenic factors, and tumor relapse was preceded by hypoxic regions in the tumors in the response phase [40]. Additionally, more invasion and metastasis was implicated as a response to this anti-angiogenic treatment, while histological analysis demonstrated a more invasive phenotype and an increase of lymph node and liver metastasis in treated mice.

Recently, a novel set of isoforms has been described, the “VEGFxxxb” isoforms, which have the same length as the classical ones, because exon 8 (present in all the formerly known isoforms) is substituted by an alternatively-spliced exon of the same size (exon 8b). Several reports have demonstrated that VEGF165b may have anti-angiogenic properties [64,65]. On the other hand, it has been suggested that these isoforms may act as VEGF receptor agonists [66,67]. We did not study the possible contribution of VEGF165b.

With regard to the results in primary cell cultures we noticed inter-individual differences, as not all cultures showed an increase in VEGF-A or GLUT-1 expression after treatment with bevacizumab. This may be a reflection of molecular differences between tumors which may activate different biochemical pathways. We may be observing adaptive or evasive resistance of the tumor cells, after an initial response phase, to adapt or evade therapy by inducing mechanisms that reduce dependence on neovascularization, leading to changed tumor proliferation. The ‘pseudohypoxic’ conditions could be responsible for selection of more malignant tumor cells, which are less sensitive to anti-angiogenic treatment and switch to alternative malignant pathways that result in proliferation, migration and invasion.

Ischemic conditions caused by anti-VEGF treatment can also lead for instance to recruitment of various bone marrow-derived cells that have angiogenic capacities. Pro-angiogenic monocytes induce vessel growth by expression of several cytokines and angiogenic factors. Research in glioblastoma multiforme has demonstrated HIF-1α to promote angiogenesis by inducing recruitment of mature F4/80+ macrophages in mice treated with bevacizumab. Additionally, a clinical study suggests that hypoxia determines survival outcome in patients treated with bevacizumab for glioblastoma multiforme [68-70]. Since it has been shown previously that malignant UM tumors in patients with a poor survival have a lot of macrophages in their tumor, this mechanism is especially relevant [71].

In conclusion, we observe an acceleration of UM growth after treatment with bevacizumab in mice. We further demonstrate a ‘pseudohypoxic’ effect, through induction of HIF-1α and VEGF-A expression, in hypoxic UM cell lines and cultures after treatment with bevacizumab. This phenomenon has been described in other tumor types and maybe the consequence of tumor adaptive or evasive resistance. The use of bevacizumab for treatment of macular edema (due to radiation retinopathy) after irradiation of UM should be considered carefully.
